# Gut microbiota prevents small intestinal tumor formation due to bile acids in gnotobiotic mice

**DOI:** 10.20517/mrr.2024.20

**Published:** 2024-08-29

**Authors:** Esther Wortmann, David Wylensek, Marijana Basic, Sven Hermeling, André Bleich, Dirk Haller, René Tolba, Gerhard Liebisch, Klaus-Peter Janssen, Thomas Clavel

**Affiliations:** ^1^Functional Microbiome Research Group, Institute of Medical Microbiology, University Hospital of RWTH Aachen, Aachen 52074, Germany.; ^2^Institute for Laboratory Animal Science and Central Animal Facility, Hannover Medical School, Hannover 30625, Germany.; ^3^Institute of Clinical Chemistry and Laboratory Medicine, University Hospital of Regensburg, Regensburg 93053, Germany.; ^4^Chair of Nutrition and Immunology, School of Life Sciences, Technical University of Munich, Freising 85354, Germany.; ^5^ZIEL - Institute for Food and Health, Technical University of Munich, Freising 85354, Germany.; ^6^Institute of Laboratory Animal Science, University Hospital of RWTH Aachen, Aachen 85354, Germany.; ^7^Technical University of Munich, School of Medicine and Health, Klinikum rechts der Isar, Department of Surgery, Munich 81675, Germany.

**Keywords:** Gut microbiota, bile acids, colorectal cancer, animal model, fecal microbiota transfer

## Abstract

**Aim:** The gut microbiota is implicated in the development of intestinal tumors. Furthermore, Western diet is a risk factor for colorectal cancer and induces alterations in both the microbiota and bile acid metabolism. Therefore, we aimed to investigate the causal role of Western diet-induced changes in the microbiota and secondary bile acid production, which were linked to disease exacerbation in *APC*^1311/+^ pigs.

**Methods:** We performed fecal microbiota transfer experiments by inoculating germfree *Apc*^1368N/+^ mice with stool from genetically engineered *APC*^1311/+^ pigs. A control group of *Apc*^1368N/+^ mice stayed germfree. All mice were fed either a control diet, or the same diet supplemented with the primary bile acid cholic acid (CA) to stimulate secondary bile acid production.

**Results:** Unexpectedly, the germfree mice fed CA had a high number of lesions in the upper small intestine, which was reduced by the colonization with microbes. The same mice (germfree, CA diet) were characterized by a remarkable lengthening of the small intestine (approximately +10 cm on average). Colonic lesions were rare and only observed in the mice that received stool from control pigs and fed the CA diet. Diversity and composition analyses showed that the microbiota transfer was incomplete. Nevertheless, mice receiving the Western diet-associated microbiota clustered separately from control animals. The effects of the CA diet on the microbiota were less pronounced and were observed primarily in mice that received stool from control pigs. Bile acid analysis in the recipient mice revealed associations between the phenotype and specific bile acid species in bile and cecum.

**Conclusion:** This descriptive study highlights the importance of diet-microbiota-bile acid interactions in intestinal morphogenesis and tumorigenesis.

## INTRODUCTION

Colorectal cancer (CRC) accounts for 9.3% of cancer-related deaths worldwide, with 1.9 million new cases and 935,000 deaths in 2020^[1]^. CRC incidence rates are highest in Europe and North America, but they are rising rapidly in Asia and Latin America^[2]^. The increase in CRC incidence has been attributed to lifestyle changes, such as increased consumption of Western diets, including higher intake of red and processed meat^[3-5]^. Human studies have shown that high-fat, low-fiber diets are associated with increased mucosal biomarkers of CRC risk^[6]^ and higher fecal levels of the secondary bile acid deoxycholic acid (DCA)^[7]^. Epidemiological data have also reported elevated levels of DCA in the stool of CRC patients^[8,9]^, but causality has not yet been established experimentally.

CRC develops over several years due to the accumulation of genetic events^[10]^. Several mouse models with shorter disease development time have been used to study CRC, many of which are based on a loss of function of the *APC* gene^[11,12]^. However, in contrast to sporadic human CRC, *Apc*^Mut^ models usually develop tumors in the small intestine^[13,14]^. While chemical treatment of wildtype mice with AOM (azoxymethane) and DSS (dextran sodium sulfate) induces tumorigenesis in the colon, acute epithelial damage and inflammation can be problematic in such models. Recently, Coleman *et al*. developed the nATF6^IEC^ genetic mouse model of colon cancer and demonstrated the causal role of microbes via microbiota transfer in germfree nATF6^IEC^ mice^[15]^. However, mice are not ideal for studying diet-disease interactions. The dietary habits and digestive physiology of pigs are closer to humans, and they still allow for tighter regulation of genetics, environmental factors, and diet composition compared to performing interventions in humans^[16]^. Previously, we found that a diet enriched in red meat and lard significantly altered fecal microbiota profiles and exacerbated disease in the genetically engineered *APC*^1311/+^ pig model of colon tumorigenesis^[17]^. However, the causal effects of microbiota changes and associated increases in fecal DCA concentrations were not tested, which can be done through fecal microbiota transfer (FMT) experiments.

Wang *et al*. found that 0.4% (w/w) of the primary bile acid, cholic acid (CA), in the diet for 16 weeks increased the number of intestinal tumors in *Apc*^Min/+^ mice^[18]^. Cao *et al*. obtained a similar result in *Apc*^Min/+^ mice when drinking water was supplemented with 0.2% DCA for 12 weeks^[19]^. The latter authors also found that normally colonized, streptomycin-treated *Apc*^Min/+^ mice receiving the microbiota from DCA-treated donors showed increased intestinal tumor numbers^[19]^. In addition, DCA and TβMCA were shown to increase intestinal stem cell proliferation and malignancy in *Apc*^Min/+^ mice^[20]^. Microbiota from human donors have also been used to test causal effects in mice. *Apc*^Min/+^ mice receiving fecal microbiota from CRC patients *vs.* healthy controls developed more intestinal tumors^[21]^. Similarly, FMT from CRC patients into antibiotic-treated or germfree C57BL/6 mice resulted in high-grade dysplasia, macroscopic polyps with AOM treatment, and a higher proportion of proliferating cells in the colon without AOM treatment^[22]^. Human-associated microbiota has been widely used to test the causality of microbiome changes in CRC and a spectrum of other diseases. However, the efficacy of transfer must be documented, and attention must be paid to the number of donors and recipients tested, as well as litter and cage effects in the case of recipients^[23]^.

Here, we sought to elucidate whether diet-induced changes in the gut microbiota and increased DCA production associated with disease exacerbation in genetically engineered *APC*^1311/+^ pigs have a causal effect on disease induction^[17]^. Therefore, we performed FMT from the pigs into germfree *Apc*^1638N/+^ mice and investigated the effects on pathology, microbiota, and bile acid metabolism.

## METHODS

### Mice

The experiment was performed under LANUV ethical approval nr. 81-02.04.2018.A425 in accordance with EU directive 2010/62/EU. *Apc*^1638N/+^ mice [B6/J.129-(Apc1638N)tm] were bred in germfree isolators (flexible film isolator type 2D, NKPisotec) under sterile conditions at the Institute of Laboratory Animal Science in the University Hospital of RWTH Aachen, Germany. The room was kept between 21-24 °C and 30%-70% humidity on a 12h:12h day:night cycle for both breeding and during the experiment.

A scheme of the experimental design is available in Supplementary Figure 1A. Germfree heterozygous *Apc*^1638N/+^ mice were transferred to sterile cages maintained in an ISOcage P-Bioexclusion System (ISO30P, Tecniplast, Italy) at the age of 4 weeks (after weaning). They were provided with autoclaved (121 °C, 20 min) standard chow diet (ssniff Spezialdiäten GmbH, cat. nr. V1534-300) and autoclaved tap water ad libitum. Fecal samples were taken to confirm the germfree status via microscopic observation after Gram-staining and cultivation on both anaerobic and aerobic agar plates for up to seven days. Mice were colonized by oral gavage with 150 µL [or max. 10% (v/w) body weight] of freshly thawed, cryopreserved (20% v/v glycerol; -80 °C) fecal microbiota from donor pigs. A second dose was administered after 72 h to favor the engraftment of strictly anoxic species. Data (phenotype, microbiota, bile acids) on the selected donor pigs are available in Supplementary Figure 2. After three weeks to reach stabilized colonization, the mice were divided into different groups that were fed with either a control diet (CD diet; ssniff Spezialdiäten GmbH, cat. nr. S5745-E902), or the same diet enriched with 0.2% (w/w) of the primary bile acid cholic acid (CA diet; ssniff Spezialdiäten GmbH, cat. nr. S5745-E903). All diets were sterilized by irradiation (2 × 25 kGy). Mice were observed and scored daily, including body conditioning, general state of health, behavior, and Bristol stool score. Body weight was recorded weekly. Mice were sampled before the end of the experiment if they reached a critical score (≥ 20 according to a predefined scoring system). All other mice were sampled at 30 weeks of age (26 weeks after colonization).

### Genotyping

Mouse tissue from ear punching was used for genotyping. DNA was extracted by incubating the tissue with 400 µL extraction buffer (1 M Tris, pH 8; 0.5 M EDTA; 5 M NaCl; 20% SDS) and 10 µL proteinase K (Carl Roth, Germany, cat. Nr. 7528.1), shaking overnight at 55 °C. After vortexing, tissue lysate (200 µL) was used for DNA precipitation by the addition of 200 µL isopropanol (Carl Roth, Germany, cat. nr. 6752.1). DNA was obtained by centrifugation (10 min, 9,600 *g*, RT), and dissolved in 200 µL TE buffer (pH 7.5), shaking overnight at 55 °C. Genotyping was done by PCR using the 2X One Taq Mastermix (New England Biolabs, cat. nr. M0482), one forward primer (5’-CAGCCATGCCAACAAAGT), and two reverse primers (5’-GGAAAAGTTTATAGGTGTCCCTTCT for wild type, 5’-GCCAGCTCATTCCTCCACTC for mutant). PCR settings were: 94 °C (60 s) initial denaturation, 30 cycles of denaturation (94 °C, 20 s), annealing (58 °C, 20 s), extension (68 °C, 20 s), and a final extension (68 °C, 60 s). Bands were observed by gel electrophoresis. Heterozygous *Apc*^1638N/+^ mice were used for the experiment.

### Sampling procedure

At 30 weeks of age (i.e., 26 weeks after colonization), mice were euthanized by isoflurane overdose (Abbvie, cat. nr. 10182054). Cardiac blood was obtained with a syringe (1010 Sterican, 27G × 0.5 inch, 0.4 × 12 mm, Braun), of which 10 µL were diluted with NaCl for red blood cell (RBC) counting using a Neubauer-improved counting chamber (0.02 mm depth). The peritoneum was examined for desmoids, and the gastrointestinal tract was removed. The length of the small intestine and colon was measured, and the intestine was then divided into duodenum, jejunum, ileum, cecum, and colon [Supplementary Figure 1B]. The weight of the spleen was recorded. Cecum and colon contents were collected separately and snap frozen. The small intestine and colon were cut open longitudinally and screened for lesions under a binocular. Potential lesions were fixed in 4% buffered formaldehyde (Otto Fischar GmbH & Co. KG, cat. nr. 27281) for 24 h and then embedded in paraffin.

### Histological analysis

A subset of paraffin-embedded intestinal lesions were stained with hematoxylin and eosin (HE) and analyzed as described previously^[24]^.

### Fecal occult blood test

The guaiac fecal occult blood test is a stool test commonly used for CRC screening in humans^[25]^. A fresh fecal pellet from individual mice was spread onto three fields of one 3-hole slide test (hemoCARE, CARE diagnostica Laborreagenzien GmbH, cat. nr. 005031-E/D). After drying, 1-2 drops of the developer solution were added and the color change was observed visually after ca. 1 min. An arbitrary score between 1-3 was given [Supplementary Figure 3].

### Bile acid analysis

Bile acids were quantified by liquid chromatography tandem-mass spectrometry (LC-MS/MS), as described previously^[26]^, with some modifications to also cover muricholic acids and their conjugates. Briefly, D4-TγMCA, D5-γMCA, D5-βMCA, D5-αMCA, D4-TβMCA, D4-TαMCA, and D5-HDCA were added to the internal standard mixture and the separation was performed on a Kinetex® 2.6 µm Biphenyl 50 × 2.1 mm column (Phenomenex, Torrance, CA, USA). Bile was diluted 500-fold prior to analysis. Cecal samples were homogenized by bead beating in 70% isopropanol and adjusted to dry weight^[27]^. Bile samples (10 nL) and cecal samples (0.1 mg dry weight) were subjected to acetonitrile precipitation. The detailed method will be described in a separate manuscript.

### 16S rRNA gene amplicon sequencing and analysis

Metagenomic DNA was isolated from cecal content and fecal samples as described before^[28]^. Amplification and sequencing of the V3-V4 regions of 16S rRNA genes were done as described previously^[29]^. Raw sequencing reads were processed using the IMNGS pipeline^[30]^, which is based on UPARSE^[31]^. The following settings were applied: max. 1 mismatch in the barcode; trimming of unpaired reads with a minimum fastq quality score of 20; min. 350 and max. 500 bp length for single reads or amplicons for paired sequences; max. 0.005 expected error rate in paired sequences; max. 50 mismatches during merging of reads; min. 70% identity of alignment during scoring merge; 20 bp trimming at forward and reverse side of the sequences; min. 0.0025 (0.25%) relative abundance cut-off (rel. abundance in at least one sample). USEARCH version 11^[32]^ was used for pairing, quality filtering, and clustering into operational taxonomic units (OTUs). Removal of non-16S sequences was done using SortMeRNA v4.2^[33]^. Sequence alignment and taxonomic classification were done with SINA version 1.6.1 and SILVA release 128^[34]^. A maximum likelihood approximation tree was calculated with Fasttree, and samples with less than 2,000 reads (9 of 85) were removed from the analysis.

The aforementioned analysis resulted in 5,484 ± 2,609 reads per sample, which were further processed using Rhea in R^[35]^. Rarefaction curves are shown in Supplementary Figure 4. To remove possible spurious taxa, the relative abundance of any SOTU or bacterial family below 0.25% was set to NA. Selected SOTUs shown in the figures were identified using EZBiocloud^[36]^. All SOTU sequences were blasted against published 7α-dehydroxylating strains [Supplementary Table 1] using 97% sequence similarity as identity cut-off. The resulting SOTU66 had 100% sequence similarity to E. muris, with an e value of 0 and a bit score of 739.

### Statistics

For statistical comparisons of microbial taxa, a prevalence cut-off of 80% across all samples was applied. For the creation of heatmaps, NAs were considered zeroes for the calculation of mean values. Heatmaps were created with the Complex Heatmap package in R^[37]^. Presence/absence of microbial taxa was tested by Fisher’s exact test in Rhea^[35]^. All other statistical analyses were done using the rstatix package in R^[38]^. Specific information on the tests used is given in the figure legends.

## RESULTS

### Cholic acid in diet-induced tumors in the upper small intestine of germfree *Apc*^1638N/+^ mice

We previously observed that an increase in the number and size of polyps in the colon of genetically engineered *APC*^1311/+^ pigs fed a Western diet (high in red meat and lard; RL diet) was associated with substantial shifts in the gut microbiota^[17]^. To investigate the causal role of these microbiota changes, we colonized germfree *Apc*^1638N/+^ mice^[14,39]^ with cryopreserved stool samples from the pigs that were fed the control diet (CTRL donors) or RL diet (RL donors) (*n* = 3 donor pigs per diet group) [Supplementary Figure 2]. An additional control group stayed germfree. All three groups of mice were then fed either a control diet (CD diet; *n* = 17 and 19 mice for CTRL and RL donors, respectively; *n* = 12 for germfree controls) or the CD diet supplemented with 0.2% (w/w) of the primary bile acid cholic acid (CA diet; *n* = 20 and 21 mice for CTRL and RL donors, respectively; *n* = 12 for germfree controls) to enhance DCA production by the microbiota.

**Figure 1:**
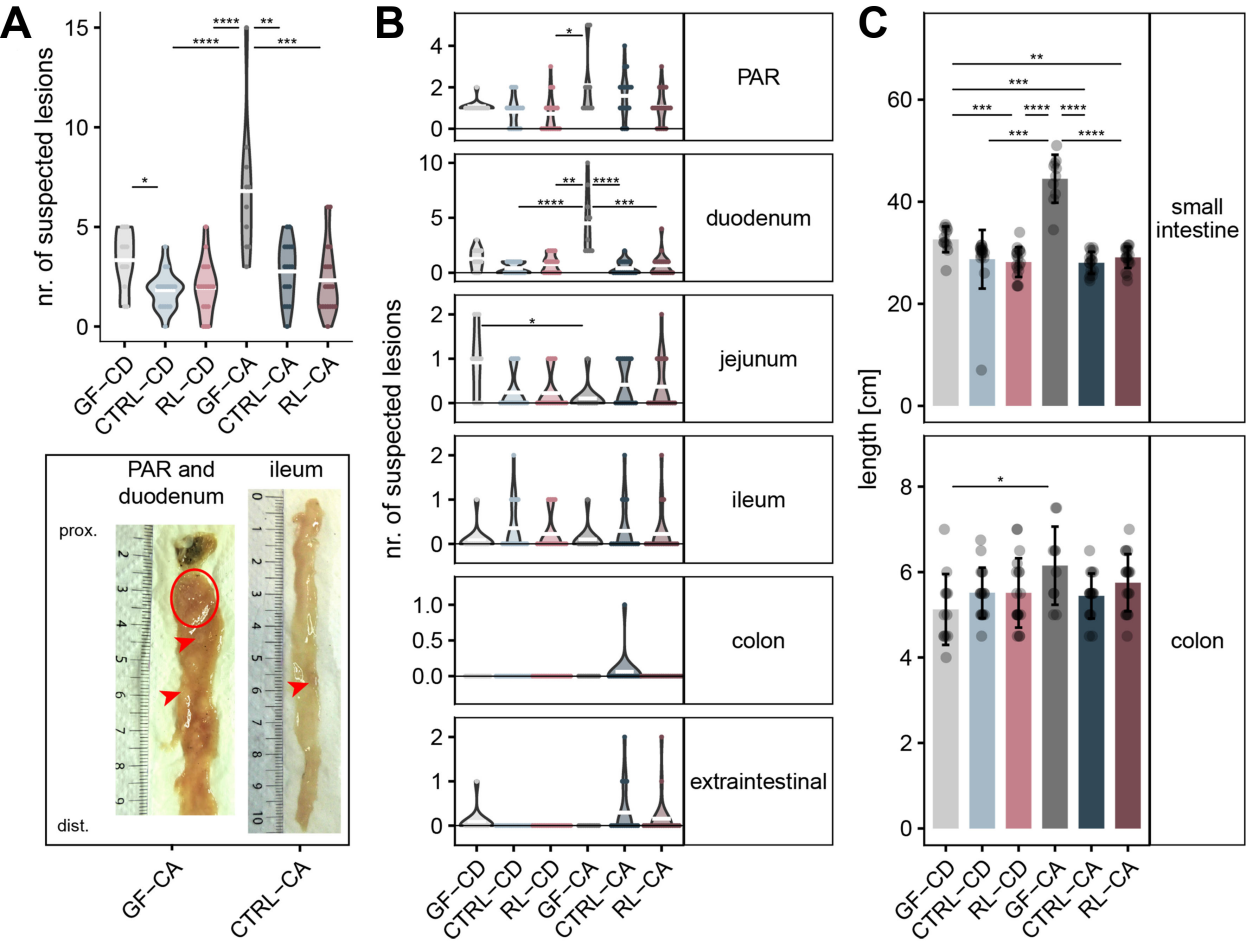
Phenotype of *Apc*^1638N/+^ mice after FMT. (A) Number of intestinal lesions for each donor-diet combination. Bottom: example macroscopic images of intestines with confirmed tumors. Left: multiple tumors (adenoma) in the PAR of a germfree mouse on the CA diet (red circle), with two additional intestinal lesions in the duodenum (red arrowheads). Right: A tumor (adenoma) in the ileum of a CTRL mouse on the CA diet (red arrowhead). Exemplary histological analyses of two lesions are shown in Supplementary Figure 5; (B) Number of lesions per gut region; (C) Length of the small intestine and colon (mean ± standard deviation). A previous version of this figure was published in the PhD thesis of Esther Wortmann (first author)^[40]^. In panels (A) and (B), the mean number of lesions is indicated by white lines. Statistics: Kruskal-Wallis followed by Dunn’s multiple comparisons with Benjamini-Hochberg adjustment (*P.adj < 0.05; **P.adj < 0.01; ***P.adj < 0.001; ****P.adj < 0.0001). FMT: Fecal microbiota transfer; PAR: periampullary region; CTRL: control donor microbiota, i.e., mice were colonized with feces from pigs fed the CTRL diet; CA: cholic acid-supplemented diet (recipient mice); GF: germfree; RL: RL donor microbiota, i.e., mice were colonized with feces from pigs fed the RL diet; CD: control diet (recipient mice).

Unexpectedly, GF mice on the CA diet were characterized by a significantly higher number of intestinal lesions compared to all colonized recipient mice [Figure 1A]. This effect was mainly driven by lesions formed in the periampullary region and in the duodenum [Figure 1B], classified as moderately dysplastic adenoma. Thus, colonization of the mice with complex porcine microbiota appeared to prevent the formation of intestinal lesions due to CA in this mouse model of tumorigenesis. In addition to the increased number of lesions, the germfree mice on the CA diet also had a significantly longer small intestine (mean length 58% higher in GF-CA *vs.* CTRL-CA) and colon (+13%) compared to colonized mice [Figure 1C]. No significant differences in dropout or fecal occult blood were observed between the groups [Supplementary Figure 6A and B]. Consistent with the higher number of lesions, germfree mice had the lowest red blood cell count, an indication of anemia and a typical sign of tumor-related morbidity in this model [Supplementary Figure 6C]. As expected, extraintestinal lesions, mainly benign desmoids in connective tissue caused by the loss of heterozygosity at the Apc locus^[14]^, remained unchanged by colonization or diet [Supplementary Figure 6D]. While body weight was highest in male mice colonized with complex microbiota and fed the CA diet [Supplementary Figure 6E], GF mice on CD were characterized by higher spleen weight, although marked inter-individual differences were observed [Supplementary Figure 6F].

While we could not validate our initial hypothesis that Western diet-induced changes in the microbiota linked to higher DCA production play a causal role in the formation of colon tumorigenesis in gnotobiotic *Apc*^1638N/+^ mice, substantial effects of microbiota-bile acid interactions were observed in the small intestine. To investigate the unexpected lack of tumors in the recipient mice, we analyzed their gut microbiota by 16S rRNA gene amplicon analysis.

### Microbiota diversity and composition differed between donor pigs and recipient mice

**Figure 2:**
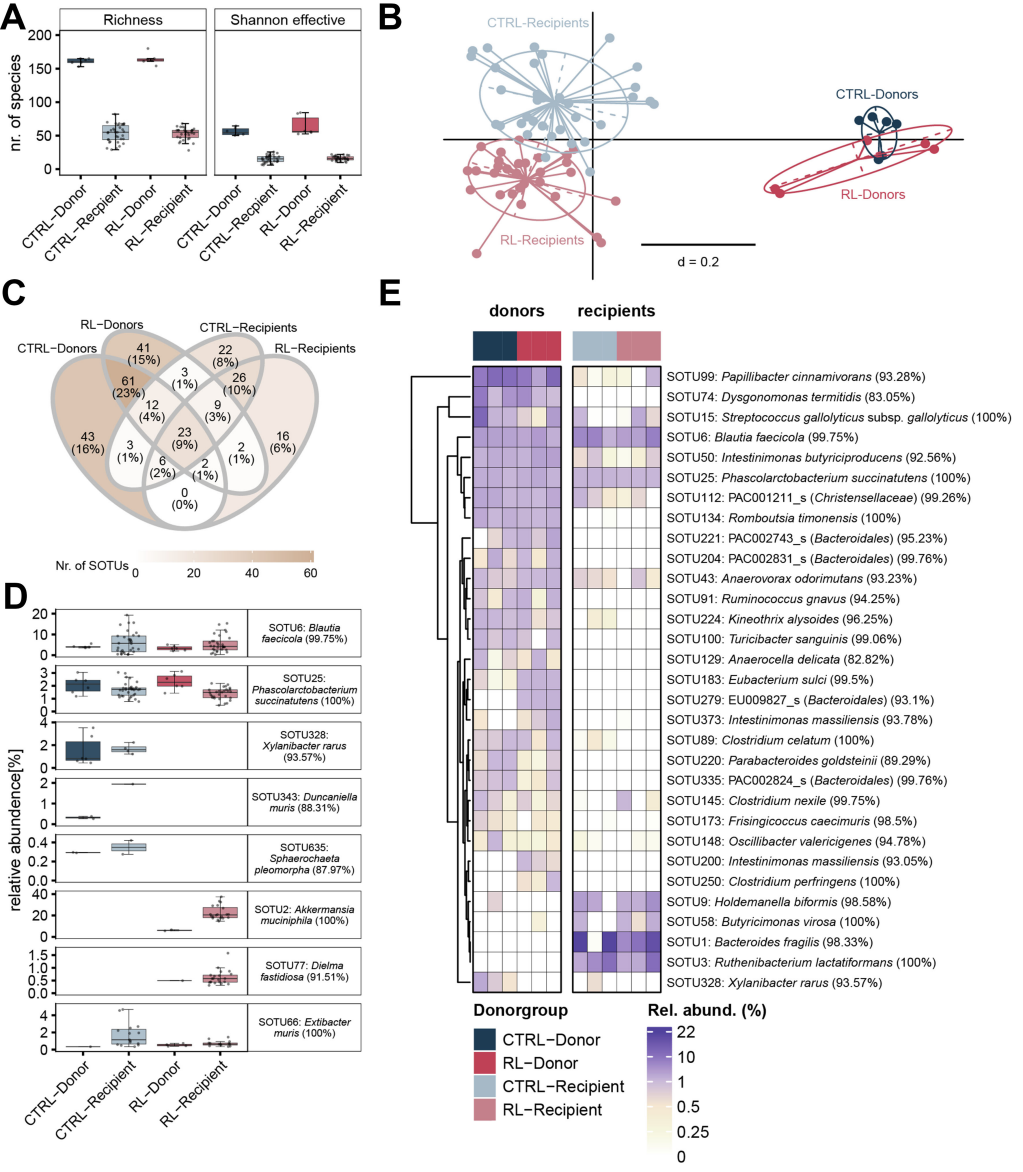
Fecal microbiota analysis using 16S rRNA gene amplicon sequencing to assess transfer efficiency. (A) Richness and Shannon effective number of species. Donor microbiota types are indicated by colors (CTRL, blue; RL, red). The different donors for each microbiota are indicated by symbols; (B) Microbiota profiles shown as NMDS, based on generalized UniFrac distances; (C) Distribution of SOTUs (Venn diagram) detected in at least one donor pig or one recipient mouse. The total number of SOTUs was 269; (D) SOTUs detected in either all groups or only one microbiota type (donors or recipients), or that matched a reference 16S rRNA gene sequence of known DCA producers. Only positive values were plotted; (E) Heatmap of SOTUs, which were characteristic of each group (prevalent in 100% of the samples per group). The columns represent the donors and corresponding recipient mice. The color in boxes indicated relative abundances as mean values. Number of sequenced samples: left, donors (dark colors), *n* = 2 cryostocks per pig; right, recipients (pale colors), *n* = 10-13 mice for CTRL microbiota, light blue, *n* = 7-13 mice for RL microbiota, light red. A previous version of this figure was published in the PhD thesis of Esther Wortmann (first author)^[40]^. CTRL: Control donor microbiota, i.e., mice were colonized with feces from pigs fed the CTRL diet; RL: RL donor microbiota, i.e., mice were colonized with feces from pigs fed the RL diet; NMDS: non-metric multidimensional scaling; SOTUs: specific molecular species; DCA: deoxycholic acid.

Comparison of both richness (total number of species) and Shannon effective counts (which take evenness into account) revealed an approximately 3-fold reduction in diversity between the stool donor inoculum (2 cryopreserved stool samples sequenced for each of the 3 donor pigs per diet) and the cecal content of recipient mice at the end of the experiment (*P* < 0.001) [Figure 2A]. Richness was slightly higher in the stool of recipient mice 3 weeks after colonization, but still much lower than in donors [Supplementary Figure 7]. The drop in diversity was accompanied by a significant shift in β-diversity, i.e., the microbiota profiles in recipient mice were clearly separated from the profiles in donor stool. Nevertheless, the two groups of recipient mice colonized with the microbiota from either CTRL or RL pigs formed separate clusters [Figure 2B]. Next, we examined the transfer of single molecular species [species-level Operational Taxonomic Units (SOTUs)]. Within the total landscape of 269 SOTUS present in at least one recipient mouse or donor inoculum, 23 (9%) were shared across all groups [Figure 2C]. An additional 21 (7%) and 13 (5%) SOTUs were shared between donors and recipients from the CTRL and RL groups, respectively. Taken together with the decrease in α-diversity [Figure 2A], and the fact that approximately 15% of all detected SOTUs were donor-specific, this confirms that species engraftment from porcine feces into the gut of germfree *Apc*^1638N/+^ mice was incomplete. A total of 22 (8%) and 16 (6%) SOTUs were detected only in recipient mice from the CTRL and RL groups, respectively [Figure 2C]. These species likely belonged to subdominant populations in the pig gut, whereas they were part of dominant communities in the mice. They contributed to the separate clustering of CTRL and RL recipients seen in the β-diversity analysis [Figure 2B], although their occurrence varied widely between individuals.

Two SOTUs were detected in all donors and recipients in both the RL and CTRL group: SOTU6 (99.5% sequence identity to *Blautia faecicola*) and SOTU25 (99.1% to *Phascolarctobacterium succinatutens*) [Figure 2D]. Three SOTUs corresponding to undescribed species were characteristic of both the donors and recipients in the CTRL group: SOTU328 (93.6% to *Xylanibacter rarus*, formerly *Prevotella rara*), SOTU343 (88.3% to *Duncaniella muris*), and SOTU635 (88.0% to *Sphaerochaeta pleomorpha*). Two SOTUs were found only in donors and recipients of the RL group: SOTU2 (100% to *Akkermansia muciniphila*) and SOTU77 (*Dielma fastidiosa*). Donor and recipient microbiota were then screened for the presence of known DCA producers. This returned only one hit, SOTU66 (100% to *Extibacter muris*), which was detected in all groups, albeit at higher relative abundances in CTRL recipient mice [Figure 2D]. Despite these few SOTUs specific to the type of microbiota transferred, the occurrence of a large fraction of dominant SOTUs was host-dependent (pig or mouse) and not due to the type of donor (pigs fed the CTRL or RL diet) [Figure 2E].

Next, we focused on the microbiota in recipient mice to specifically investigate differences due to the donor microbiota type and the diet (all mice were fed either a control diet or a diet supplemented with the primary bile acid CA).

### The microbiota in recipient mice was more influenced by donor than by diet

**Figure 3:**
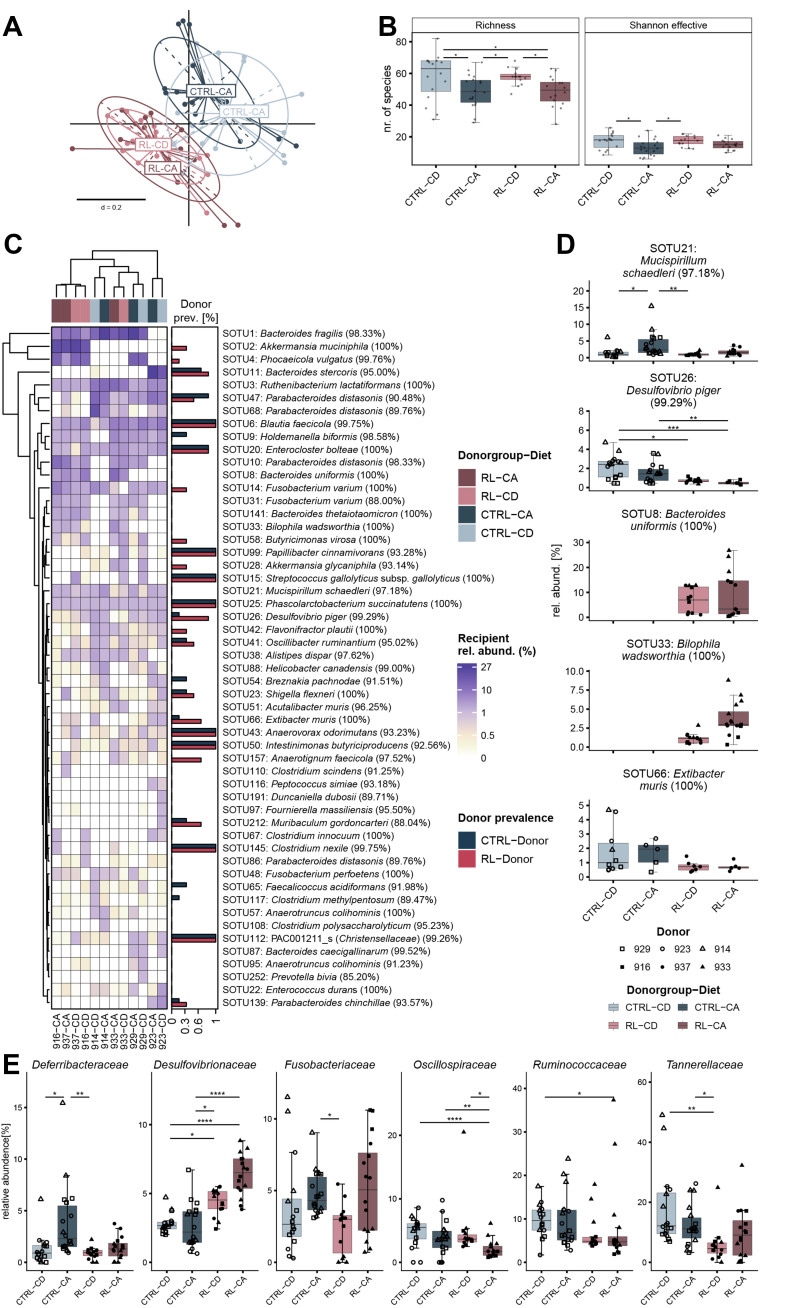
Microbiota profiles in recipient mice colonized with different porcine stool samples (CTRL or RL donors; blue and red, respectively) and fed either CD or CA diet (light and dark colors, respectively). (A) Generalized Unifrac distances shown as NMDS plot (PERMANOVA *P*.adj = 0.057); (B) Richness and Shannon effective counts for each donor-diet combination; (C) Comparative analysis of dominant SOTUs (mean relative abundance > 1% in at least one group). The columns, each representing a donor-diet combination, were clustered by Euclidean distance. The prevalence of the given SOTUs in the donor inoculum is indicated by bars on the right side. The closest taxonomic hit (based on EZBiocloud) of each SOTU is indicated by species name and percentage of sequence identity in parentheses; (D) Prevalent and abundant SOTUs (present in > 80% of all recipient mice; rel. abund. > 1% in at least one group) highlighting differences between the groups; (E) Major differences in the occurrence of dominant bacterial families. The criteria for selection/testing were the same as in (D). Statistics: Kruskal Wallis with Dunn’s multiple comparison, Benjamini-Hochberg correction (*P.adj < 0.05; **P.adj <0.01; ***P.adj < 0.001; ****P.adj < 0.0001). A previous version of this figure was published in the PhD thesis of Esther Wortmann (first author)^[40]^. CTRL: Control donor microbiota, i.e., mice were colonized with feces from pigs fed the CTRL diet; RL: RL donor microbiota, i.e., mice were colonized with feces from pigs fed the RL diet; CD: control diet (recipient mice); CA: cholic acid-supplemented diet (recipient mice); NMDS: non-metric multidimensional scaling.

After a 3-week stabilization period post-colonization, all recipient mice were placed on either a control diet (CD diet) or the same diet supplemented with 0.2% (w/w) of the primary bile acid CA (CA diet). A comparison of the microbiota structure revealed a clustering primarily due to donor type, but also showed that the CTRL recipients were more affected by the CA diet than the RL recipients [Figure 3A and Supplementary Figure 8]. This was reflected by lower richness and Shannon effective counts in CTRL recipient mice fed the CA diet [Figure 3B]. Euclidean clustering of abundant SOTUs (> 1% relative abundance in at least one group), plotted using the average occurrence across all mice for each donor-diet combination (*n* = 12; 2 donor microbiota types, 3 donor pigs each, 2 recipient diets), confirmed that the donor microbiota type (CTRL, blue *vs.* RL, red) had a stronger effect on microbiota composition compared to the recipient diet (light *vs.* dark colors), although inter-individual differences between donors were observed. Recipients of RL donors 916 and 937 clustered separately (far left of the dendrogram), which was mainly due to SOTU2 (*Akkermansia muciniphila*, 100%) and SOTU4 (*Phocaeicola vulgatus*, 99.8%; formerly *Bacteroides vulgatus*) [Figure 3C]. Statistical comparison of prevalent and abundant SOTUs (detected in > 80% of all mice and at a relative abundance of > 1% in at least one donor-diet group) revealed significant differences for SOTU21 (*Mucispirillum schaedleri*, 97.2%), which was highest in CTRL recipients on CA diet [Figure 3D]. In contrast, SOTU26 (*Desulfovibrio piger*, 99.3%) was highest in CTRL recipients on CD diet and only very low abundant in RL recipients [Figure 3D]. SOTU8 (*Bacteroides uniformis*, 100%) and SOTU33 (*Bilophila wadsworthia*, 100%) were detected only in RL recipients, and SOTU33 was significantly elevated in the RL mice fed the CA diet [Figure 3D]. SOTU66, which matched the 16S rRNA gene sequence of the DCA-producing species *Extibacter muris*, had a higher relative abundance in CTRL recipients, but this was independent of diet [Figure 3D]. At the level of dominant bacterial families, *Desulfovibrionaceae* were significantly increased in RL recipients on the CA diet [Figure 3E], primarily due to the presence of *B. wadsworthia*. In contrast, *Deferribacteraceae* were significantly increased in CTRL recipients on the CA diet, reflecting the increase in *M. schaedleri*. Other bacterial families, such as *Oscillospiraceae*, *Ruminococcaceae*, or *Tannerellaceae*, were more influenced by the donor microbiota type than by diet [Figure 3E].

In summary, the transfer of bacterial taxa from porcine donor microbiota into germfree *Apc*^1368N/+^ was partial, which may have contributed to the lack of tumor induction in the distal gut. Differences in diversity and composition were observed due to donor microbiota type and, in specific cases, due to diet. However, as all colonized mice fed CA had fewer small intestinal lesions and a shorter intestine compared to their germfree counterparts, the observed microbiota differences were irrelevant to the phenotype [Figure 1]. Because of our original goal to study bile acid metabolism by the microbiota and the CA-induced phenotype seen in germfree mice, we next measured bile acid levels in the mouse cecum and bile.

### Colonization and diet-induced changes in bile acids

**Figure 4:**
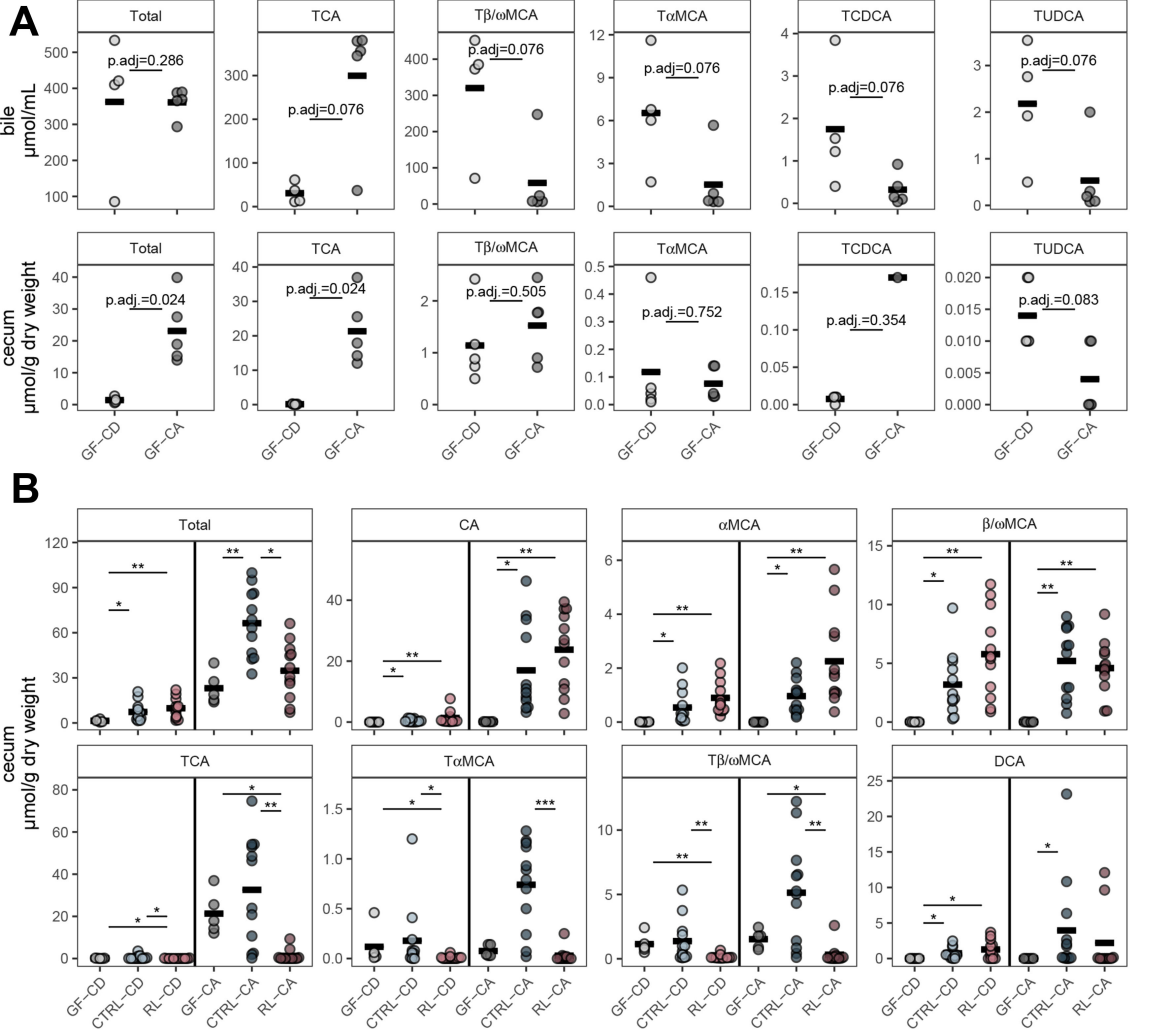
Bile acid levels in the gallbladder and cecum of germfree and gnotobiotic *Apc*^1368N/+^ mice. (A) Concentrations of dominant bile acid species (mean concentration per group > 0.5 µmol/mL) in the gallbladder (top) and corresponding values in the cecum (bottom) of germfree mice; (B) Effects of colonization and diet (CA supplementation) on bile acid levels in the mouse cecum. The data for germfree mice are the same as in panel a (bottom row). βMCA and ωMCA could not be distinguished by the analysis and are shown as β/ωMCA. Statistics: (A) comparison of diet groups in germfree mice: Wilcoxon rank-sum test with Benjamini-Hochberg adjustment for multiple comparisons; (B) comparison of microbiota groups for each diet: Kruskal-Wallis with Benjamini-Hochberg correction for multiple comparisons followed by Dunn’s test for pairwise comparisons (*P.adj < 0.05; **P.adj < 0.01; ***P.adj < 0.001). CA: Cholic acid-supplemented diet (recipient mice).

Due to the unexpected phenotype in the upper small intestine of germfree mice in combination with bile acid supplementation in the diet, bile acids were measured in the bile. Total bile acid levels in the gallbladder were similar between germfree mice on the control diet (CD) and CA diet [Figure 4A, top row]. In contrast, there were significant differences for individual bile acids: in germfree mice on the CA diet, taurocholic acid (TCA) accounted for most biliary bile acids, whereas bile from mice on the CD diet contained mostly tauro-β/ω-muricholic acid (Tβ/ωMCA, Figure 4A, top row). Diet effects in the cecum of germfree mice included significantly higher levels of total bile acids when the diet was supplemented with CA, mainly due to markedly elevated levels of TCA [Figure 4A, bottom row].

When comparing bile acids in the cecum of germfree and colonized mice [Figure 4B], microbial colonization increased total bile acid levels, with significantly higher values in mice colonized with the CTRL microbiota and fed the CA diet. Although added to the diet, CA was not abundant in the cecum of germfree mice, suggesting it is rapidly absorbed in the small intestine and conjugated to TCA in the liver, which then cannot be deconjugated in the intestine due to the absence of bile salt hydrolase (BSH) from the microbiota. Only low levels of conjugated bile acids were detected in the cecum of RL recipient mice, suggesting higher BSH activity within their microbiota. All colonized mice were characterized by: (i) increased levels of muricholic acid isomers; and (ii) as expected, detection of the secondary bile acid DCA. DCA levels appeared to be slightly elevated in CTRL *vs.* RL recipients on the CA diet, reflecting the higher relative abundance of SOTU 66 (100% similarity to the DCA producer *E. muris*) observed in CTRL mice [Figure 3D].

To conclude, the small intestinal phenotypes observed in germfree mice fed CA were associated with high TCA and low Tβ/ωMCA levels in bile compared to their counterparts on control diet, and with low levels of unconjugated bile acids (CA, αMCA, β/ωMCA) and absence of the secondary bile acid DCA in the cecum compared to colonized mice. Further mechanistic studies are needed to understand how the microbiota regulates bile acid metabolism to influence CA-induced tumor burden and small intestinal lengthening.

## DISCUSSION

Diet composition influences CRC risk, the gut microbiota, and bile acid metabolism. In this study, we investigated whether Western diet-induced alterations of the gut microbiota in the *APC*^1311/+^ pig model of colorectal adenomas have deleterious effects upon transfer in gnotobiotic *Apc*^1368N/+^ mice.

CA in the diet (0.2% w/w, 23 weeks) stimulated lesion formation in the upper small intestine (periampullary region and duodenum) of germfree *Apc*^1368N/+^ mice. Previous work has reported conflicting results on the effects of primary bile acids in female *Apc*^min/+^ mice, both in terms of the colonization status and gut region of interest. Mahmoud *et al*. found that a 10-week dietary intervention with 0.5% chenodeoxycholic acid, which is the other main primary bile acid besides CA in human but is only produced in small amounts in the mouse liver due to further conversion to muricholic acids (MCAs), increased the number of lesions in the duodenum of conventionally colonized mice^[41]^. Wang *et al*. showed that CA in the diet (0.4%, 12 weeks) increased the number of lesions, but primarily in the middle and distal small intestine^[18]^. Importantly, CA-induced lesion formation was reduced by microbial colonization in our model. This occurred regardless of the recipient mouse diet (with or without CA) or the type of microbiota used for colonization (i.e., from pig fed the control or Western diet), indicating that the protective role of microbes in CA-induced duodenal carcinogenesis is provided even by clearly distinct microbial communities. This is at odds with the data of Wang *et al*. that CA-induced intestinal carcinogenesis in the distal small intestine of colonized female *Apc*^min/+^ mice was abrogated after microbiota perturbation by treatment with an antibiotic cocktail (ampicillin, vancomycin, neomycin, metronidazole)^[18]^. Collectively, this suggests that microbiota regulation of bile acid metabolism may be protective in the upper but not the distal small intestine.

Another observation in our experiments was the substantial lengthening of the small intestine of germfree *Apc*^1368N/+^ mice fed the CA diet. Previous work has reported small intestinal lengthening in germfree mice compared to colonized counterparts^[42,43]^, but the effects of bile acids have not been studied. Small intestinal lengthening also occurred in germfree *Apc*^1368N/+^ mice on the control diet (significantly compared to mice colonized with the RL-microbiota), but this was substantially enhanced by CA in the diet. Recently, Nguyen *et al*. reported that elevated levels of CA in the gut lumen due to intestine-specific deletion of the bile acid transport regulator called small heterodimer partner (SHP) were associated with changes in villus length and goblet cell number in the ileum of male mice fed a 1% CA diet for 5 days^[44]^. Small intestine length was not assessed, but this finding suggests that CA is involved in molecular processes underlying tissue morphogenesis.

In the germfree *Apc*^1368N/+^ mice, supplementation of the diet with CA shifted the biliary bile acid composition from Tβ/ωMCA to TCA. In conventional mice, the ratio of TCA to TMCA was reported to be approx. 2:1, while in germfree mice, the ratio was 1:1^[45]^. In the germfree *Apc*^1368N/+^ mice on CA diet, the TCA to Tβ/ωMCA ratio was as high as 14:1, suggesting that the bile acid pool of these mice was out of balance. Interestingly, the colonized mice on CA diet had ratios of approx. 6:1 (CTRL recipients) and 3:1 (RL recipients), indicating that colonization normalized the bile acid balance. The ratio of CA to MCA species plays an important role in determining the hydrophobicity of the bile acid pool in mice and consequently affects cholesterol absorption^[45,46]^. The unusually high levels of TCA in the bile and intestine of germfree *Apc*^1368N/+^ mice on CA diet, due to the absence of microbially produced BSH, may, therefore, have led to increased cholesterol absorption and impaired cell proliferation. It may also be directly related to the tumor formation observed in germfree mice, as the upper small intestine is directly exposed to secreted bile, which is known to have DNA-damaging effects^[47]^. This may indicate that tumors in the upper intestinal tract, which are clinically relevant in human FAP syndrome, have a different etiology than in CRC^[48]^. The increased intestinal length in the germfree mice on the control diet may be due to other reasons. TβMCA is an FXR antagonist and was found to promote the proliferation of Lgr5+ stem cells^[20]^. TCA, on the other hand, is an FXR agonist and was found to enhance the proliferation of mouse intestinal cells^[49]^. The production of secondary bile acids such as DCA in the distal gut of colonized mice may also have had indirect effects in the upper regions of the intestine.

This study is the first to examine the effects of fecal microbiota transplantations from pigs into mice in the context of dietary interventions and CRC. The incomplete efficacy of the microbiota transfers may have been due to altered microbial viability in the cryopreserved samples. In addition, differences in diet, bile acid pool composition, or intestinal physiology of the hosts may have influenced the survival of certain species. For example, mice predominantly produce tauro-conjugated bile acids, while most bile acids in the pig liver are conjugated to glycine^[50,51]^. Furthermore, SOTUs detected only in mice but not in the donor pigs may have been present in their gut, but at low relative abundances and thus undetectable by amplicon sequencing (e.g., < 0.25% relative abundance). Despite partial microbiota engraftment, we observed interesting effects of the microbiota type and diet fed to the recipients. SOTU33 (*Bilophila wadsworthia*, 100%) and SOTU8 (*Bacteroides uniformis*, 100%) were only detected in RL recipients. Both *Bacteroides* and *Bilophila* species are resistant to bile and were found to be increased after intake of an animal-based diet in humans^[7]^. Growth of *B. wadsworthia* was shown to be promoted by TCA due to the further metabolism of taurine^[52]^. Combined metagenomic and metabolomic analysis in the stool of individuals with CRC reported a positive association between the occurrence of *B. wadsworthia* and DCA levels^[9]^. However, this species does not encode a bai operon in its genome. As the RL recipient mice in our FMT trial did not have more intestinal lesions, the increase in *B. wadsworthia* observed in these mice had no detrimental effects on the host. The CTRL recipients were characterized by higher levels of the secondary bile acid DCA, consistent with higher levels of SOTU66 (100% to *E. muris*). The significantly lower levels of tauro-conjugated bile acids in the cecum of RL recipients compared to CTRL recipients suggest a higher capacity of their microbiota to deconjugate bile acids. The higher total amount of bile acids in the cecum of CTRL recipients suggests that these mice had lower bile acid absorption in the small intestine, which in turn affected their cecal microbiota. Our study shows that it is imperative to analyze the microbiota of donors and recipients in detail to avoid misleading interpretation of results from FMT experiments, as emphasized by others previously^[53]^.

This study has obvious limitations. It is a descriptive work and the molecular mechanisms underlying the phenotype observed in germfree mice fed bile acids are not provided. Furthermore, although we took care to perform a well-designed study in gnotobiotic mice with a sufficient number of mice, taking into account litter and cage effects, and using a colonization protocol usually appropriate to enable the engraftment of strictly anaerobic bacteria in the intestine, the efficacy of microbiota transfer was not optimal, which prevented us from drawing a clear conclusion on our initial hypothesis.

In conclusion, microbiota transfer from *APC*^1311/+^ pigs did not induce colon tumors in gnotobiotic *Apc*^1368N/+^ mice. In contrast, CA induced lesions in the duodenum and elongation of the small intestine under germfree conditions. This phenotype was associated with changes in TCA and Tβ/ωMCA levels in bile and with the absence of secondary bile acid production such as DCA. Further work is required to investigate the underlying molecular mechanisms and potential clinical implications.

## DECLARATIONS

### Availability of data and materials

The 16S rRNA gene amplicon dataset was deposited at ENA and is available under the following project number: PRJEB73361.

### Copyright

© The Author(s) 2024.
