# Supplementary material for: Gut microbiota prevents small intestinal tumor formation due to bile acids in gnotobiotic mice
Source: Microbiome Res Rep. 2024 Aug 29;3(4):44. doi: 10.20517/mrr.2024.20 (PMC11684917; doi:10.20517/mrr.2024.20)
Supplement: Supplementary file 1 [file mrr-3-4-44-SupplementaryMaterials.pdf]

Wortmann *et al. Microbiome Res Rep* 2024;3:44
DOI: 10.20517/mrr.2024.20

Microbiome Research Reports

Supplementary Materials

Gut microbiota prevents small intestinal tumor formation due to bile acids in gnotobiotic mice

Esther Wortmann[1], David Wylensek[1], Marijana Basic[2], Sven Hermeling[3], André Bleich[2], Dirk Haller[4,5], René Tolba[6], Gerhard Liebisch[3], Klaus-Peter Janssen[7], Thomas Clavel[1]

[1]Functional Microbiome Research Group, Institute of Medical Microbiology, University Hospital of RWTH Aachen, Aachen 52074, Germany.

[2]Institute for Laboratory Animal Science and Central Animal Facility, Hannover Medical School, Hannover 30625, Germany.

[3]Institute of Clinical Chemistry and Laboratory Medicine, University Hospital of Regensburg, Regensburg 93053, Germany.

[4]Chair of Nutrition and Immunology, School of Life Sciences, Technical University of Munich, Freising 85354, Germany.

[5]ZIEL - Institute for Food and Health, Technical University of Munich, Freising 85354, Germany.

[6]Institute of Laboratory Animal Science, University Hospital of RWTH Aachen, Aachen 85354, Germany.

[7]Technical University of Munich, School of Medicine and Health, Klinikum rechts der Isar, Department of Surgery, Munich 81675, Germany.

Correspondence to: Prof. Thomas Clavel, Functional Microbiome Research Group, Institute of Medical Microbiology, University Hospital of RWTH Aachen, Pauwelsstraße 30, Aachen 52074, Germany. E-mail: tclavel@ukaachen.de

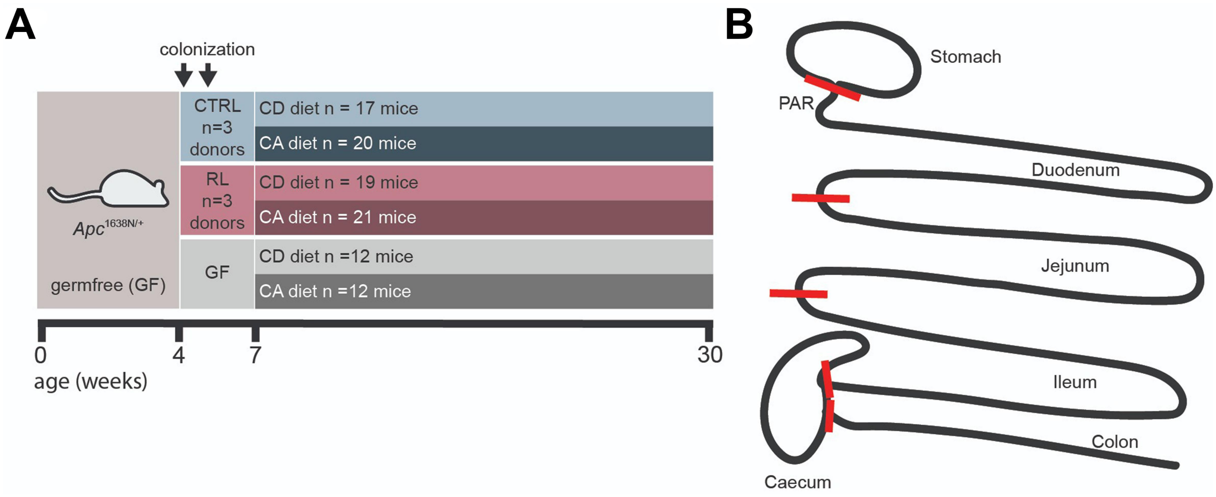

Supplementary Figure 1. Experimental design and sampling information. (A) Simplified scheme of the experimental design, including timeline and number of donor pigs and recipient mice; (B) Gut regions as defined for cutting during sampling.

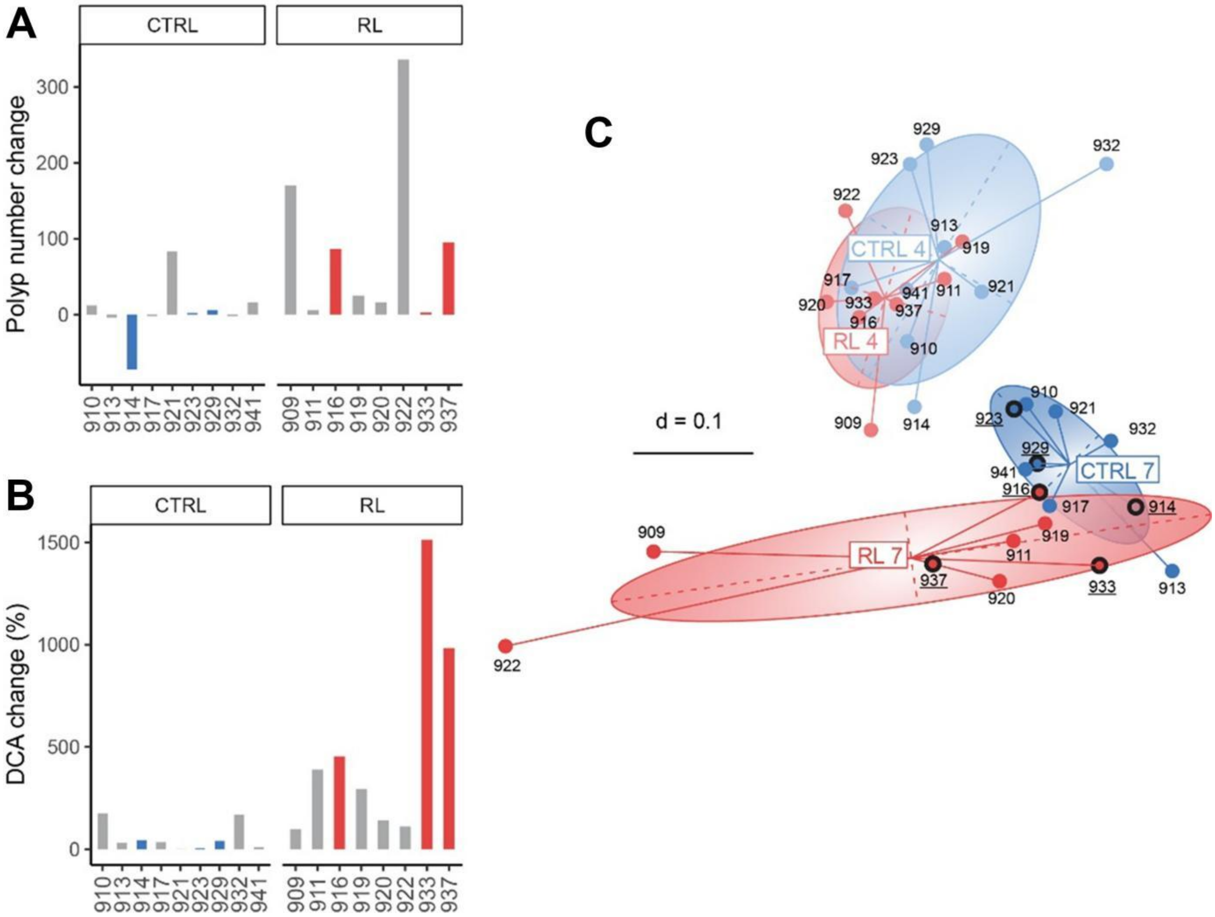

Supplementary Figure 2. Information on the selected pigs that served as donors for fecal microbiota transfer (*n* = 3 for each diet). (A) Change in polyp number in the distal colon; (B) Change in fecal DCA levels over the feeding period (7 months vs. 4 months); (C) Microbiota profiles, shown as NMDS plots based on Generalized UniFrac distances. The pigs selected for FMT are marked in color (a and b) or with underscore and black circles (C). Colors: blue = pigs fed the control diet (CTRL); red = pigs fed the diet supplemented with red meat and lard (RL). Light colors indicate the 4-month time point (before intervention). Dark colors indicate the 7-month time point (after intervention).

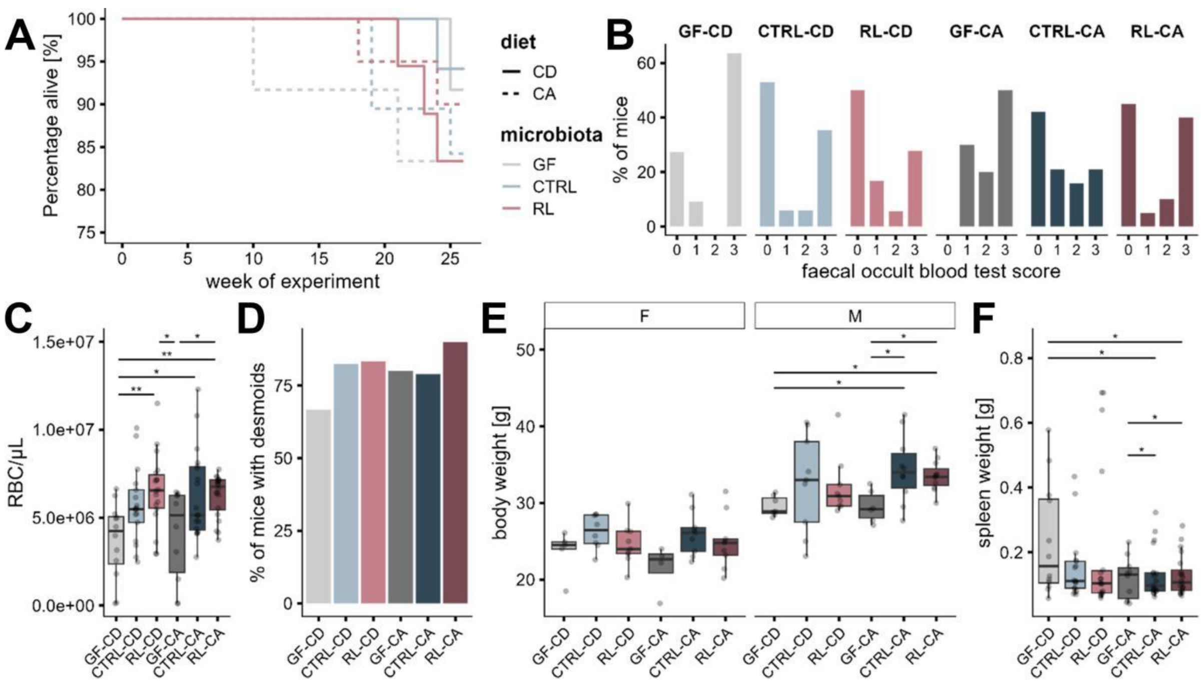

Supplementary Figure 3. Additional sampling data. (A) Kaplan-Meier survival curves (log-rank test, $P = 0.9$); (B) Percentage of mice assigned to the different fecal occult blood test scores; (C) Red blood cell (RBC) count; (D) Percentage of mice with desmoids; (E) Body weight over time; (F) Spleen weight. Statistics in (C): Kruskal-Wallis followed by Dunn's multiple comparisons with Benjamini-Hochberg adjustment (*P.adj < 0.05; **P.adj < 0.01). GF, germfree; CTRL, recipient mice colonized with the microbiota from donor pigs fed a control diet; RL= recipient mice colonized with the microbiota from donor pigs fed an experimental diet enriched in red meat and lard; CD, control diet (recipient mice); CA, control diet supplemented with the primary bile acid cholic acid (recipient mice).

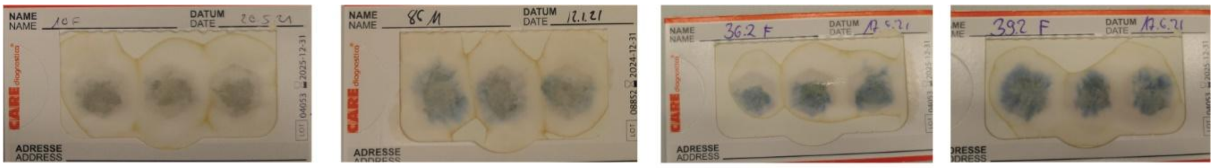

FOB score 0 FOB score 1 FOB score 2 FOB score 3

Supplementary Figure 4. Arbitrary scoring scheme for Fecal Occult Blood (FOB) tests.

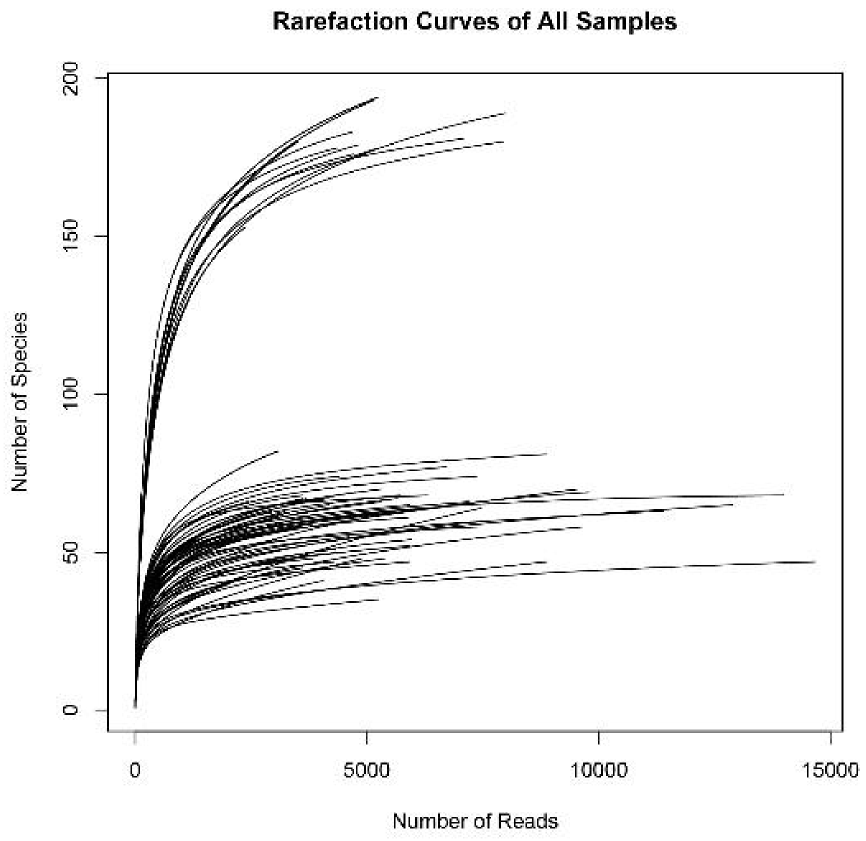

Supplementary Figure 5. Rarefaction curves of donor samples (top lines) ad recipient mice (bottom lines).

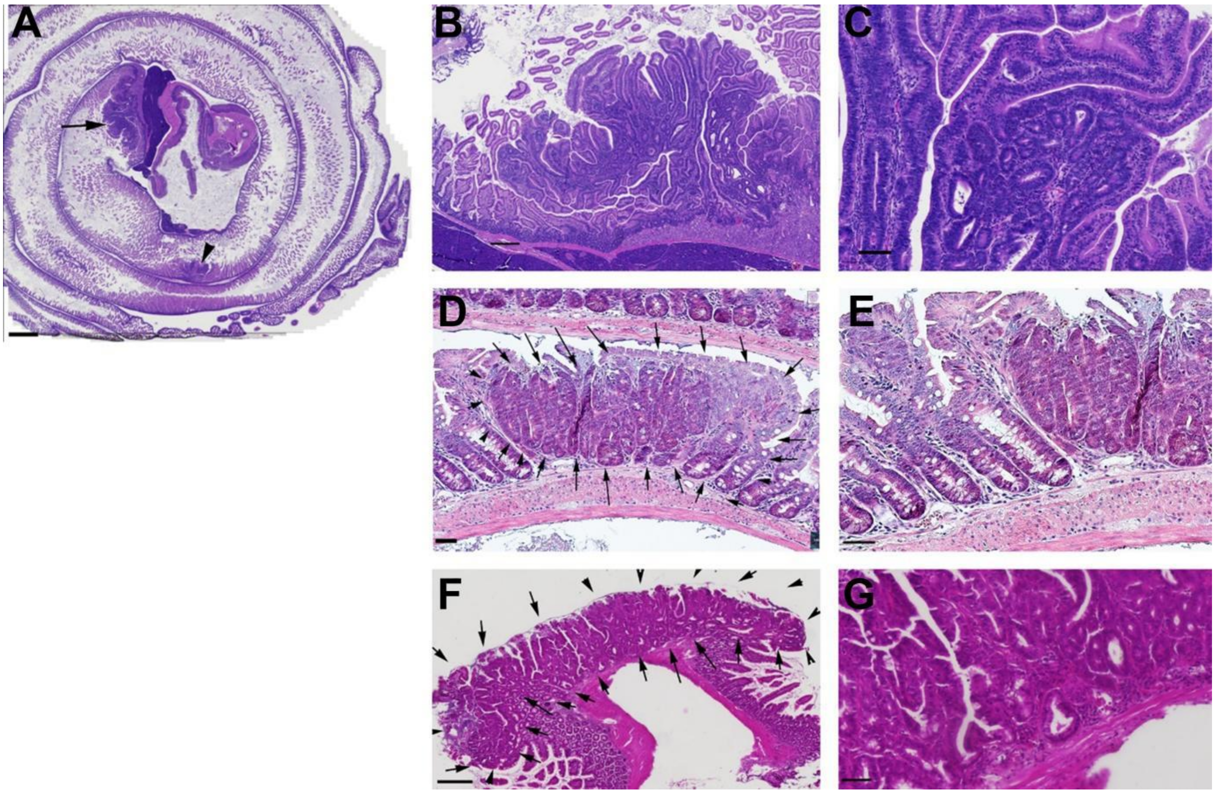

Supplementary Figure 6. Exemplary HE staining of tumors shown in Figure 1. (A) Overview (Swiss roll) of gut tissue from a germfree $Apc^{1638N/+}$ mouse (female, 7 months of age) fed autoclaved standard chow diet; bar = 1 mm; arrow shows a tumor in the periampullar region (PAR); arrowhead shows a tumor in the duodenum. (B, C) adenoma in the PAR of a germfree mouse fed the CA diet; (B) Overview, bar = 250 µm; note the presence of pancreatic tissue at the bottom; (C) high power field of view of dysplastic area, bar = 50 µm; (D, E) Adenoma in the jejunum of a gnotobiotic mouse (CTRL microbiota) fed the CA diet; d Overview, arrows denote the outline of the lesion, bar = 250 µm. e High power field of view, bar = 50 µm; (F, G) adenoma with low grade dysplasia in the duodenum of germfree mouse fed the CTRL diet. (D) Overview, bar = 250 µm. (E) High power field of view, bar = 50 µm.

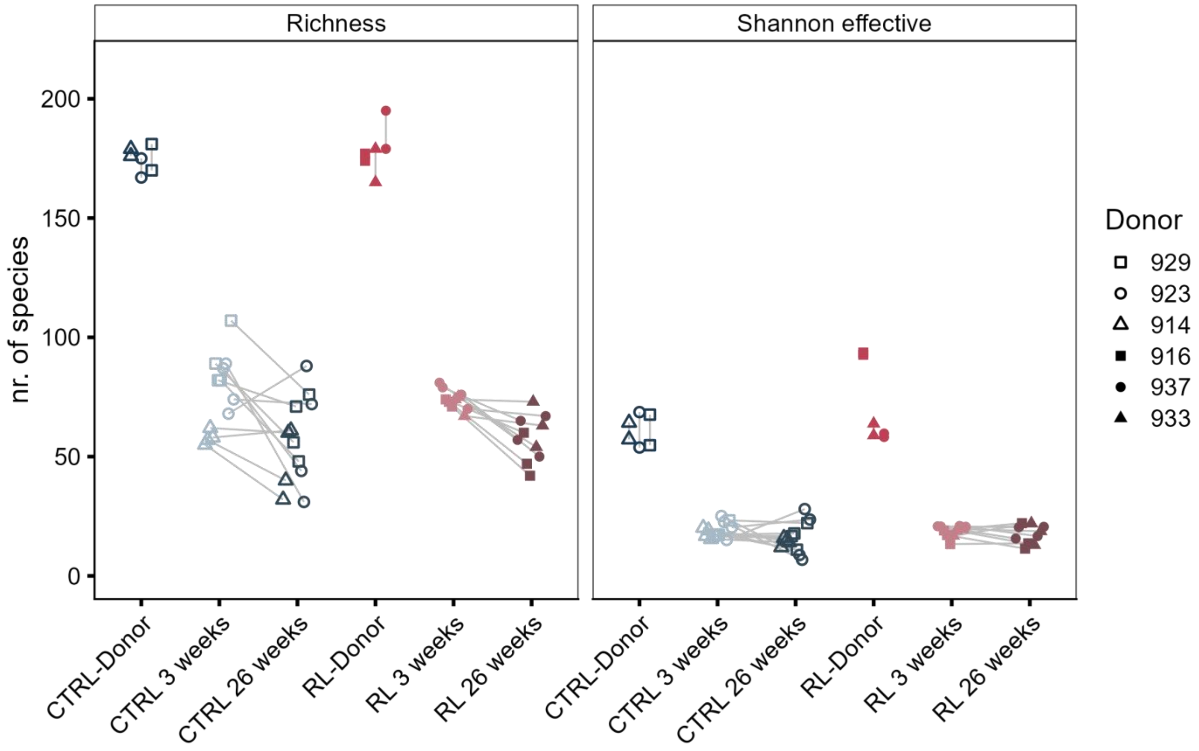

Supplementary Figure 7. Richness (left plot) and Shannon effective counts (right plot) over time in donor samples (pig stool), mouse feces 3 weeks after colonization (light colors), and cecal content at the end of the experiment, i.e., 36 weeks after colonization (dark colors). The donors are indicated with different symbols. Colors: blue, CTRL microbiota; red, RL microbiota. Samples from the same mouse/donor are connected with grey lines.

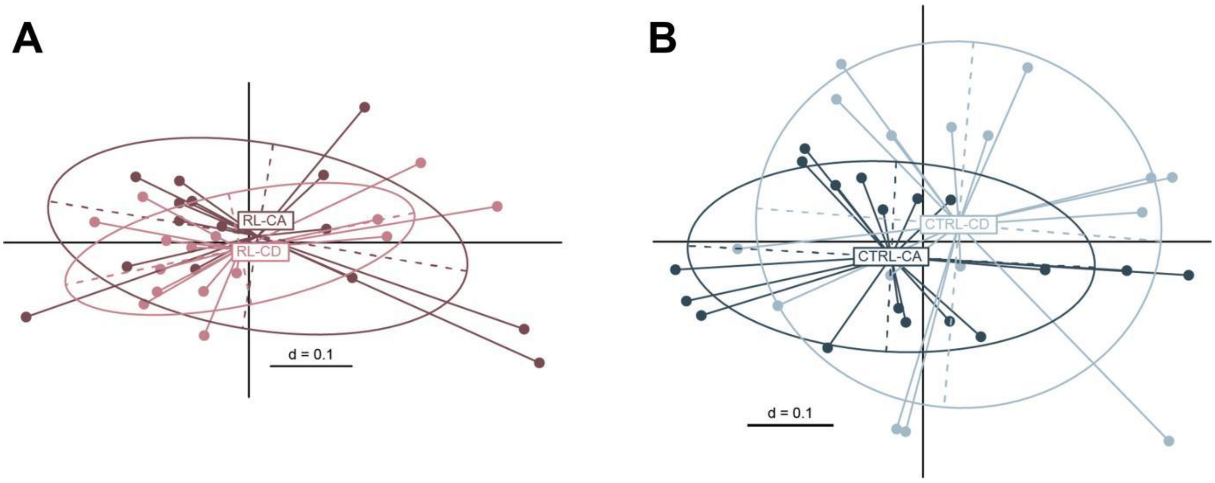

Supplementary Figure 8. Pairwise comparisons of microbiota structure, shown as NMDS plots. (A) Comparison of diet effect within RL-recipient mice; PERMANOVA, *P*.adj = 0.057. (B) Comparison of diet effect within CTRL-recipient mice; *P*.adj = 0.017.

Supplementary Table 1. Published species/strains with a bai operon

Species	Strain	Accession nr.	Ref.
Clostridium scindens	ATCC35704	ABFY02000057	[1]
Clostridium [Extibacter] hylemonae	TN-271	AB023972	[2]
Clostridium leptum	DSM753	ABCB02000019	[1]
Clostridium sordelli	ATCC9714	NR_112173.1	[3]
Peptacetobacter hiranonis	TO-931	AB023970	[4,5]
Proteocatella sphenisci	PPP2	AF450134	[6]
Extibacter muris	DSM8650^{T}	KR364761	[7]

References

1. Doerner KC, Takamine F, LaVoie CP, Mallonee DH, Hylemon PB. Assessment of fecal bacteria with bile acid 7α-dehydroxylating activity for the presence of bai-like genes. Appl Environ Microbiol. 1997;63(3):1185–8.
2. Ridlon JM, Kang DJ, Hylemon PB. Isolation and characterization of a bile acid inducible 7α-dehydroxylating operon in Clostridium hylemonae TN271. Anaerobe. 2010;16(2):137–46.
3. Takamine F, Imamura T. Isolation and Characterization of Bile Acid from Human Feces. Microbiol Immunol. 1995;39(1):11–8.
4. Wells JE, Hylemon PB. Identification and characterization of a bile acid 7α-dehydroxylation operon in Clostridium sp. strain TO-931, a highly active 7α-dehydroxylating strain isolated from human feces. Appl Environ Microbiol. 2000 Mar;66(3):1107–13.
5. Kitahara M, Takamine F, Imamura T, Benno Y. Clostridium hiranonis sp. nov., a human intestinal bacterium with bile acid 7α-dehydroxylating activity. Int J Syst Evol Microbiol. 2001;51(1):39–44.
6. Kim KH, Park D, Jia B, Baek JH, Hahn Y, Jeon CO. Identification and Characterization of Major Bile Acid 7α-Dehydroxylating Bacteria in the Human Gut. mSystems. 2022;7(4).
7. Streidl T, Karkossa I, Segura Muñoz RR, Eberl C, Zaufel A, Plagge J, et al. The gut bacterium Extibacter muris produces secondary bile acids and influences liver physiology in gnotobiotic mice. Gut Microbes. 2021;13(1):1–21.
